# High-resolution ultrasound of the annular pulley system in the toes: sonographic anatomy and pathological cases

**DOI:** 10.1007/s00256-025-04901-w

**Published:** 2025-03-22

**Authors:** Federico Pistoia, Marta Macciò, Maria Elena Susi, Riccardo Picasso, Federico Zaottini, Giovanni Marcenaro, Simone Rinaldi, Maribel Miguel Perez, Antonio Quaglio, Matteo Formica, Carlo Martinoli

**Affiliations:** 1 IRCCS Ospedale Policlinico San Martino, Largo Rosanna Benzi, 10, Genoa, Italy; 2 Department of Health Sciences (DISSAL), Radiology Section, University of Genova, Via Pastore 1, Genoa, Italy; 3 Departamento de Patología y Terapéutica Experimental, Facultad de Medicina y Ciencias de La Salud (Campus de Bellvitge), Unidad de Anatomía y Embriología Humana, Universitat de Barcelona, Barcelona, Spain; 4 Department of Integrated Surgical and Diagnostic Sciences (DISC), University of Genova, Via Pastore 1, Genoa, Italy

**Keywords:** Ultrasound, High resolution, Pulley, Toes

## Abstract

**Objective:**

To validate high-frequency ultrasound as a valuable imaging modality in the assessment of the annular pulley system of the toes, describing their normal sonographic appearance and presenting some illustrative pathological cases.

**Materials and methods:**

The first phase of this observational study involved examining the annular pulley system of the flexor tendons of the toes in a series of cadaveric specimens, testing the ability of ultrasound to recognize these structures. In the second phase, two expert sonographers examined a sample of 15 healthy adult participants using two different 18–5 MHz and 24–8 MHz linear array ultrasound probes. Sonographic visibility, position, and maximal thickness of each annular pulley were assessed.

**Results:**

In the cadaveric study, ultrasound provided identification of 4 annular pulleys in the lesser toes and 3 in the great toe across 8 feet from 4 cadavers. In 30 feet of 15 healthy participants, ultrasound was able to consistently recognize and measure the annular pulleys of toes I–IV, with minimal variability between operators. The V toe annular pulleys were less reliably visualized. The intraclass correlation coefficient (ICC) analysis of pulley maximal thickness measurements between the two sonographers showed moderate agreement for the 24–8 MHz probe with a mean ICC of 0.393 (CI: 0.315–0.471) and better agreement for the 18–5 MHz probe with a mean ICC of 0.529 (CI: 0.442–0.616).

**Conclusion:**

This study provides a comprehensive description of the sonoanatomy of the annular pulleys of the flexor tendons in the toes and demonstrates the potential of ultrasound as a valuable tool for diagnosing pathologies of these structures.

## Introduction

Numerous studies have explored the role of ultrasound (US) in the evaluation of the flexor pulley system of the fingers and its accuracy in diagnosing different pathological conditions such as trigger finger. However, the US appearance of the flexor pulley system of the toes has never been described. To date, only a few studies have been published on the anatomical and imaging appearance of the flexor tendons of the toe pulleys, despite the existence of documented pathology affecting this pulley system [1, 2]. In particular, the only imaging study describing the normal appearance of the flexor pulleys of the toes was performed in cadaveric toe specimens on an ultrahigh field 11,7 Tesla (T) MR [2], while no studies have investigated the feasibility of conventional imaging modalities, including US, in the evaluation of these pulleys. The aim of this study was to validate high-frequency US as a valuable imaging modality in the assessment of the annular pulley system of the toes, describing their normal sonographic appearance and presenting some emblematic pathological cases affecting the annular pulleys of the toes, and resembling the pathology commonly encountered in the annular pulleys of the fingers.

## Materials and methods

This study was approved by the local Ethics Committee (Registry number CER Liguria 222/2024-DB id 13,881), and informed consent was obtained from all the participants. The first phase of the study involved examining the annular pulley system of the flexor tendons of the toes in a series of cadaveric specimens. The ability of US to recognize the different annular pulleys of the toes was tested by injecting colored latex or positioning marking pins under US guidance in the cadaveric toe specimens. After the anatomical study, two sonographists (F.P. and M.M.) with specific skills in musculoskeletal US examined a sample of 15 adult participants enrolled in December 2024. The US machine was equipped with a high-frequency 18–5 MHz hockey-stick transducer and a 24–8 linear array probe (Aplio i800, Canon Medical System, Ōtawara, Japan). The inclusion criteria were based on the absence of any history of relevant pathology or surgical procedures on the feet. In every participant, all toes of the feet were examined. The volar aspect of each toe was scanned both longitudinally and transversely, and the complete sonographic examination took approximately 40 min per participant. Sonographic visibility, position, and maximal thickness of each annular pulley were assessed.

### Statistical analysis

This is an observational study. No prespecified sample size calculation was performed, as all data were derived from measurements obtained during the imaging sessions. Mean, median, standard deviation, and ranges were calculated for thickness measurements. Intraclass correlation coefficients (ICC) with 95% confidence intervals were used to assess agreement between sonographers for each probe type and annular pulley. Statistical analyses were performed using R (version 4.4.2).

## Results

### Cadaveric study

**Fig. 1:**
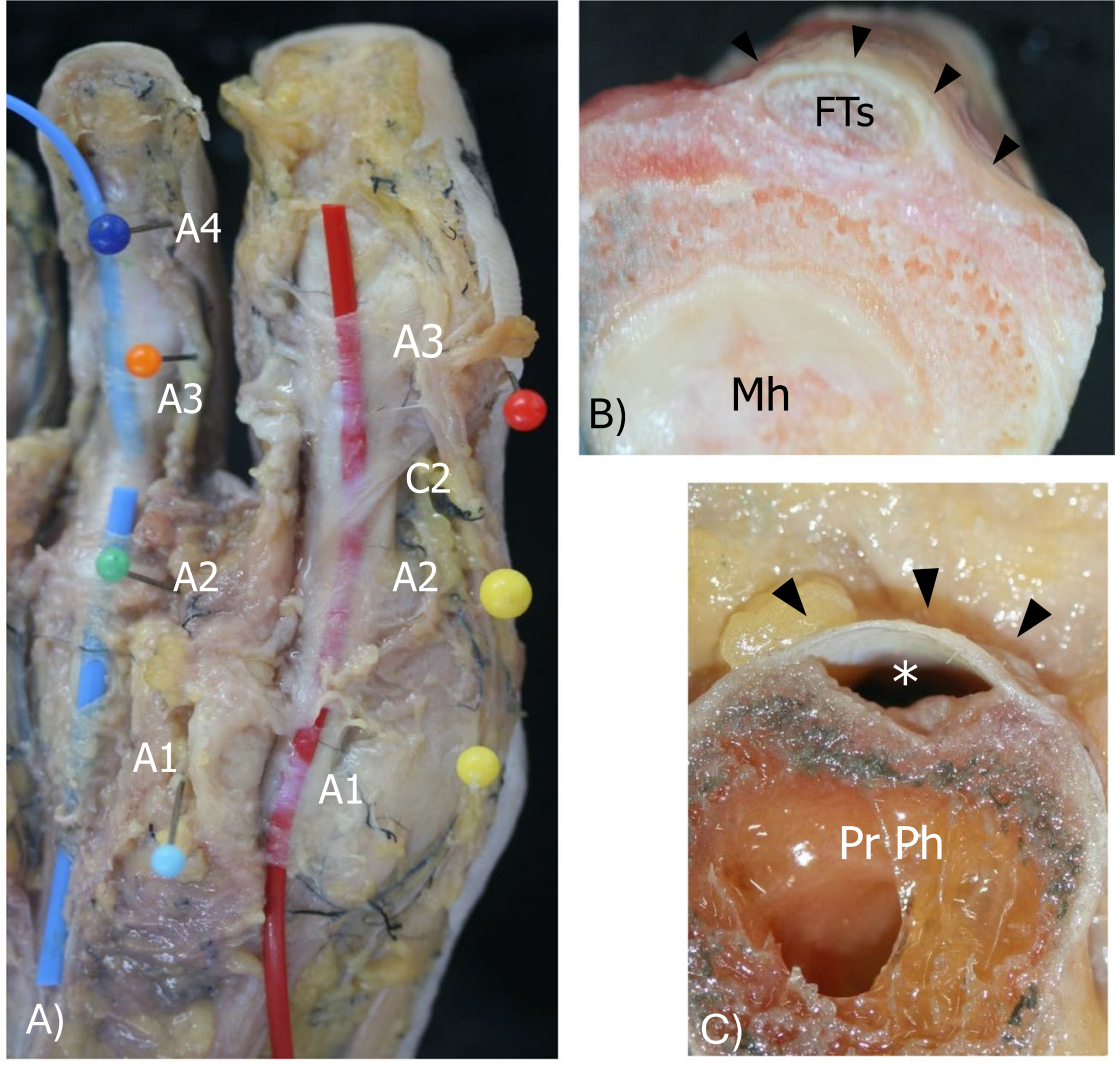
Cadaveric dissection of the great toe and II toe. **A** Coronal image of the dissected great toe and II toe. Marking pins were placed under US guidance at the level of the three annular pulleys (A1, A2, and A3) in the great toe and the four annular pulleys (A1, A2, A3, and A4) in the second toe. To improve visualization of the annular pulleys, two thin tubes—red for the great toe and blue for the second toe—were positioned beneath the pulleys and over the flexor tendons. Note the C2 pulley in the great toe. **B** Axial view of the first annular pulley (black arrowheads) of the hallux, overlying the flexor hallucis longus and brevis tendon (FTs) at the level of the first metatarsal head (I MH). **C** Axial view of the second annular pulley (black arrowheads) of the hallux at the level of the proximal phalanx of the hallux (Pr Ph) after removal of the flexor hallucis longus tendon (*). A1, first annular pulley; A2, second annular pulley; A3, third annular pulley; A4, fourth annular pulley; C2, second cruciform pulley; FTs, flexor tendons; Mh, metatarsal head; Pr Ph, proximal phalanx

**Fig. 2:**
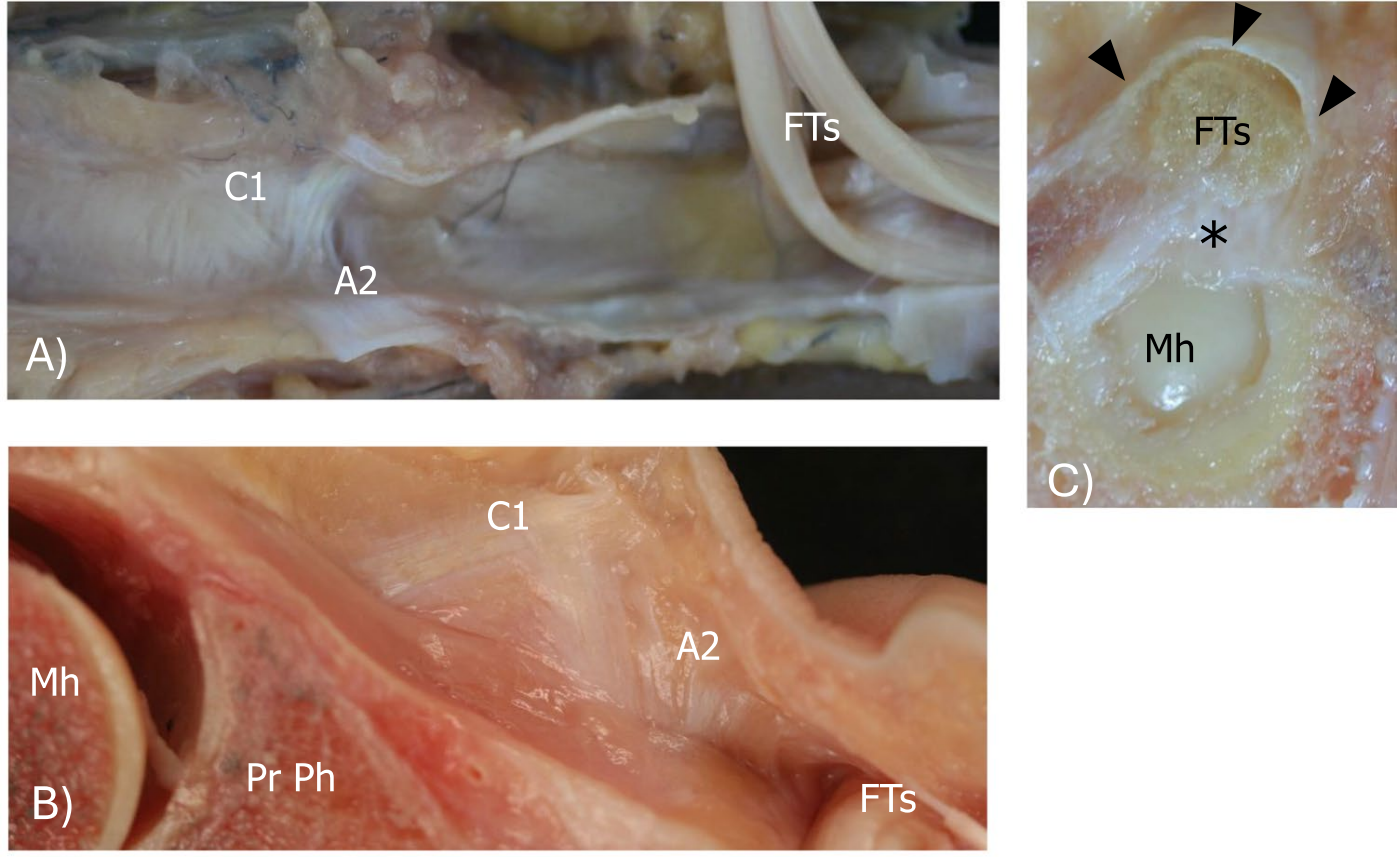
Cadaveric dissection of the II and III toe. **A** Coronal image of a dissected second toe following the elevation of the flexor tendons (Fl Ts), highlighting the close anatomical relationship between the ventral surface of the second annular pulley (A2) and the first cruciform pulley (C1). **B** Sagittal image of a dissected third toe showing the first cruciform pulley (C1) and the second annular pulley (A2) after the elevation of the flexor tendons (FTs). **C** Axial image of the first annular pulley (black arrowheads) of the second toe, overlying the flexor tendons (Fts) at the level of the second metatarsal head (Mh). Note the volar plate beneath the Fts. C1, first cruciform pulley; A2, second annular pulley; FTs, flexor tendons; Mh, metatarsal head; Pr Ph, proximal phalanx

The cadaveric study was performed on 8 cadaveric feet harvested from 4 fresh frozen cadavers. The cadavers did not have any history of relevant pathology or surgical procedures on the feet. The specimens were thawed at room temperature immediately prior to the study, and cadaver arteries were filled with silicone to enhance their visualization during dissection. On all feet and prior to dissection, a musculoskeletal radiologist (F.P.) identified with US the presumed annular pulleys along the ventral side of the toes. The localization procedures, performed under direct real-time US visualization, were based in two feet on injection of diluted colored latex adjacent to the presumed annular pulleys and in the other feet on positioning marking pins. Each injection was performed using a US-guided in-plane approach, targeting the presumed annular pulleys with a 22 Gauge, 1.5 inch (38 mm) stainless steel needle. In each specimen, 0.2–0.3 mL of diluted, blue, or green latex (50% water, 50% latex) was injected. Then, an experienced anatomist (M.M.P) carefully dissected each specimen to evaluate if the latex or the marking pins were centered over the annular pulleys. Of the 8 cadaveric feet, 2 feet were cut in the sagittal plane and 2 others in the transverse plane. On anatomical dissection, 4 annular pulleys were most identified in the lesser toes and 3 annular pulleys in the great toe, along with 2 cruciform pulleys found in both the great and lesser toes (Figs. 1 and 2). In all cadaveric specimens, the annular pulleys were located as identified on US. In lesser toes, the first annular pulley (A1) was located at the level of the metatarsophalangeal joint, the second annular pulley (A2) at the proximal phalanx, the third annular pulley (A3) at the proximal interphalangeal joint, and the fourth annular pulley (A4) at the distal interphalangeal joint, while the first cruciform pulley (C1) was identified between A1 and A2, and the second cruciform pulley (C2) between A2 and A3. In the great toe, A1 was located at the level of the metatarsophalangeal joint, A2 at the proximal phalanx, and A3 at the distal interphalangeal joint, while C1 was identified between A1 and A2, and C2 between A2 and A3.

### Sonographic study

**Fig. 3:**
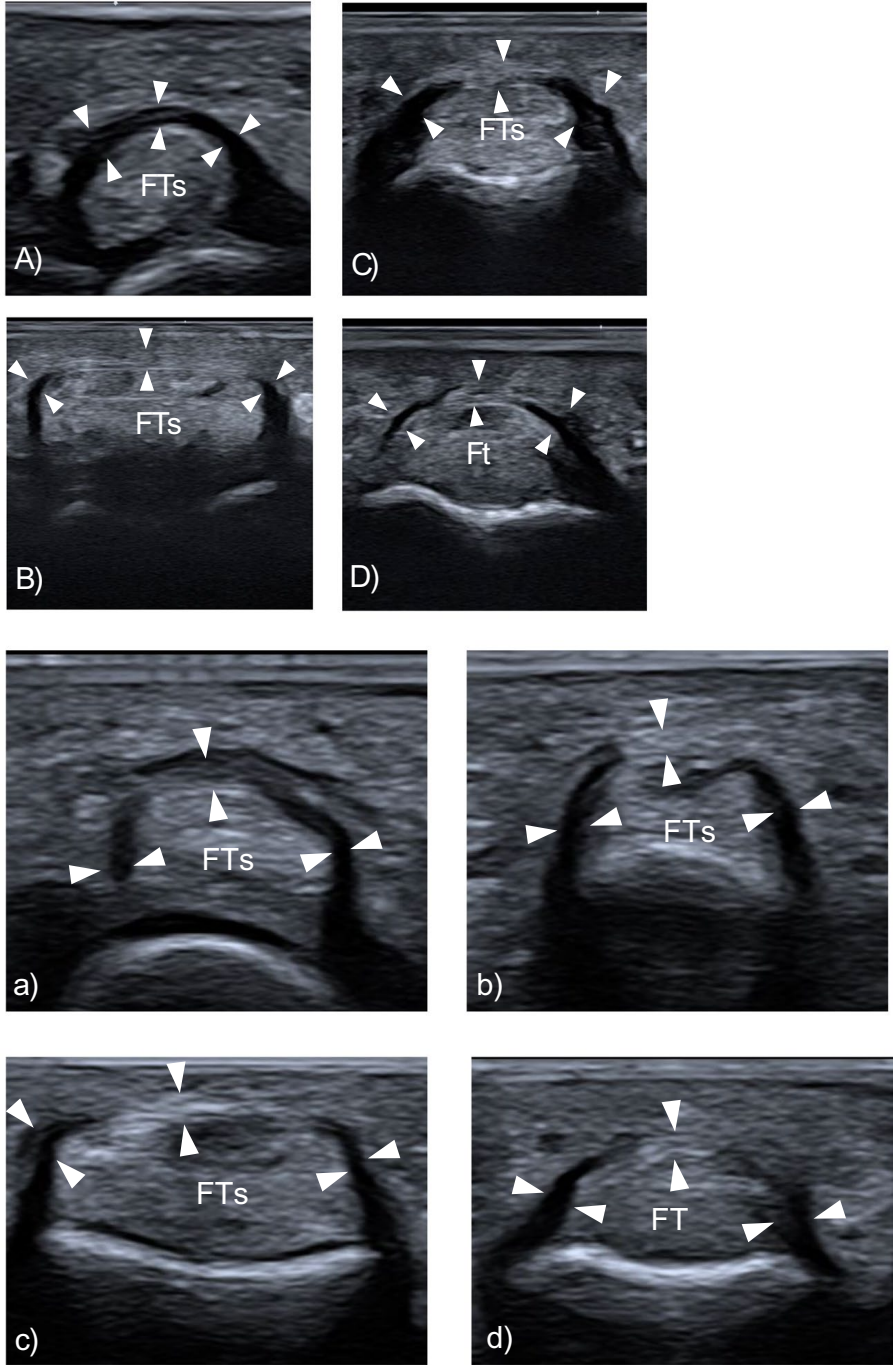
Normal ultrasound appearances of annular pulleys in healthy individuals. Transverse US images of the annular pulleys of the II toe obtained with a 22–8 MHz hockey-stick probe (A) and an 18–5 MHz probe (a). **A**-**a** A1 pulley (arrowheads) overlying the flexor tendons (FTs) at the level of the metatarsophalangeal joint. **B**-**b** A2 pulley (arrow-heads) overlying the flexor tendons (FTs) at the level of the proximal phalanx. **C**–**c** A3 pul- ley (arrowheads) overlying the flexor tendons (FTs) at the level of the proximal interphalangeal joint. **D**-**d** A4 pulley overlying the flexor digitorum longus tendon (Ft) at the level of the distal interphalangeal joint. FTs, flexor tendons; FT, flexor tendon

After the cadaveric study, a consecutive series of 15 Caucasian healthy participants, 7 females, ranging in age from 26 to 63 years (mean ± SD: 27 ± 13) were examined. The volar aspect of each toe on both feet was scanned longitudinally and transversely to assess the visibility and position of the pulleys. The pulleys thickness was measured only on the transverse plane using both 24-8 and 18-5 MHz linear array probes. On US, the annular pulleys appeared as slightly hyperechoic structures prone to anisotropy (Fig. 3). Since reliable sonographic identification of the two cruciform pulleys was not possible, to ensure accurate measurement and avoid possible misattribution, the thickness of the A1 and A2 pulleys was measured on their distal edge. The annular pulleys of toes I through IV were consistently visible and measurable, with minimal variability between operators. In contrast, the annular pulleys of the V toe were less reliably visualized. Tables 1 and 2 detail the mean and median maximal annular pulley thickness measurements obtained using the 24–8 MHz and 18–5 MHz probes, respectively, along with the total number of visible annular pulleys for each toe, as assessed by both operators. The intraclass correlation coefficient (ICC) analysis of pulley thickness measurements between the two sonographers showed moderate agreement for the 24–8 MHz probe with a mean ICC of 0.393 (CI: 0.315–0.471), and better agreement for the 18–5 MHz probe with a mean ICC of 0.529 (CI: 0.442–0.616). Although the ICC may not indicate strong agreement, the mean difference between observers was only 0.04 ± 0.01 mm, which is extremely small and likely not clinically relevant given the mean thickness of the annular pulleys (0.4-0.5 mm). Table 3 presents the ICC between the two operators for each toe and probe type. Table 4 compares the pulley thickness measurements by toe, side, probe, and sonographer. In two healthy participants peri-pulley ganglion cysts were noted.

**Table 1:** Pulley thicknesses obtained with the 24–8 MHz probe. Mean, median, standard deviation (SD), interquartile range (Q1, Q3), and sample size (*N*) of maximal pulley thickness measurements (in mm) for each toe (I to V) on the right (R) and left (L) feet. Values in parentheses represent 95% confidence intervals. The sample size of the V toe is smaller compared to the other toes, reflecting the less consistent visualization of its annular pulleys

Toe	Sonographist	Mean (mm)	Median (mm)	SD	Q1	Q3	*N*
I-L	1	0.48 (0.46, 0.49)	0.48 (0.46, 0.5)	0.055	0.44	0.52	45
I-L	2	0.48 (0.46, 0.49)	0.49 (0.47, 0.51)	0.055	0.44	0.52	45
I-R	1	0.48 (0.46, 0.5)	0.48 (0.46, 0.5)	0.065	0.44	0.51	45
I-R	2	0.47 (0.46, 0.49)	0.47 (0.46, 0.48)	0.047	0.44	0.50	45
II-L	1	0.46 (0.44, 0.47)	0.46 (0.44, 0.48)	0.058	0.41	0.5	60
II-L	2	0.44 (0.43, 0.46)	0.44 (0.43, 0.45)	0.044	0.41	0.48	60
II-R	1	0.46 (0.44, 0.47)	0.46 (0.44, 0.47)	0.046	0.42	0.48	60
II-R	2	0.45 (0.44, 0.46)	0.44 (0.43, 0.45)	0.047	0.42	0.47	60
III-L	1	0.46 (0.45, 0.48)	0.46 (0.45, 0.48)	0.055	0.42	0.49	60
III-L	2	0.43 (0.42, 0.44)	0.43 (0.42, 0.44)	0.045	0.40	0.46	60
III-R	1	0.45 (0.44, 0.47)	0.45 (0.44, 0.46)	0.053	0.42	0.49	60
III-R	2	0.44 (0.43, 0.46)	0.44 (0.43, 0.45)	0.041	0.41	0.47	60
IV-L	1	0.45 (0.44, 0.46)	0.45 (0.43, 0.47)	0.051	0.41	0.49	59
IV-L	2	0.44 (0.43, 0.45)	0.43 (0.42, 0.44)	0.041	0.41	0.47	60
IV-R	1	0.45 (0.44, 0.46)	0.45 (0.43, 0.47)	0.052	0.41	0.49	59
IV-R	2	0.44 (0.43, 0.45)	0.44 (0.43, 0.46)	0.044	0.42	0.47	60
V-L	1	0.41 (0.38, 0.45)	0.4 (0.36, 0.43)	0.072	0.38	0.48	18
V-L	2	0.42 (0.4, 0.44)	0.42 (0.4, 0.44)	0.042	0.40	0.45	15
V-R	1	0.43 (0.41, 0.45)	0.43 (0.41, 0.45)	0.054	0.39	0.46	27
V-R	2	0.42 (0.41, 0.44)	0.41 (0.4, 0.42)	0.037	0.40	0.45	23

**Table 2:** Pulley thicknesses obtained with the 18–5 MHz probe. Mean, median, standard deviation (SD), interquartile range (Q1, Q3), and sample size (*N*) of maximal pulley thickness measurements (in mm) for each toe (I to V) on the right (R) and left (L) feet. Values in parentheses represent 95% confidence intervals. The sample size of the V toe is smaller compared to the other toes, reflecting the less consistent visualization of its annular pulleys

Toe	Sonographist	Mean (mm)	Median (mm)	SD	Q1	Q3	*N*
I-L	1	0.51 (0.49, 0.52)	0.5 (0.48, 0.52)	0.054	0.47	0.55	43
I-L	2	0.5 (0.48, 0.52)	0.5 (0.48, 0.52)	0.062	0.46	0.55	45
I-R	1	0.52 (0.5, 0.54)	0.5 (0.47, 0.53)	0.074	0.46	0.57	45
I-R	2	0.51 (0.48, 0.53)	0.49 (0.46, 0.52)	0.081	0.46	0.55	45
II-L	1	0.49 (0.48, 0.5)	0.48 (0.47, 0.5)	0.057	0.45	0.52	60
II-L	2	0.48 (0.46, 0.49)	0.46 (0.45, 0.48)	0.058	0.43	0.51	60
II-R	1	0.5 (0.48, 0.51)	0.49 (0.48, 0.5)	0.054	0.46	0.52	60
II-R	2	0.49 (0.47, 0.5)	0.47 (0.45, 0.49)	0.058	0.44	0.52	60
III-L	1	0.49 (0.47, 0.5)	0.49 (0.48, 0.5)	0.060	0.45	0.51	60
III-L	2	0.47 (0.45, 0.48)	0.46 (0.45, 0.47)	0.050	0.43	0.49	60
III-R	1	0.48 (0.46, 0.49)	0.48 (0.47, 0.49)	0.057	0.45	0.5	60
III-R	2	0.47 (0.46, 0.49)	0.46 (0.45, 0.48)	0.061	0.45	0.5	60
IV-L	1	0.47 (0.46, 0.49)	0.47 (0.46, 0.48)	0.046	0.45	0.5	59
IV-L	2	0.46 (0.45, 0.47)	0.45 (0.43, 0.47)	0.051	0.42	0.5	60
IV-R	1	0.48 (0.47, 0.49)	0.48 (0.47, 0.49)	0.047	0.45	0.5	59
IV-R	2	0.47 (0.46, 0.48)	0.48 (0.47, 0.49)	0.054	0.43	0.5	60
V-L	1	0.43 (0.39, 0.47)	0.44 (0.4, 0.48)	0.080	0.4	0.5	14
V-L	2	0.45 (0.42, 0.47)	0.45 (0.43, 0.47)	0.053	0.43	0.48	13
V-R	1	0.45 (0.43, 0.47)	0.44 (0.42, 0.45)	0.054	0.41	0.46	24
V-R	2	0.43 (0.42, 0.45)	0.44 (0.42, 0.46)	0.037	0.41	0.45	17

**Table 3:**
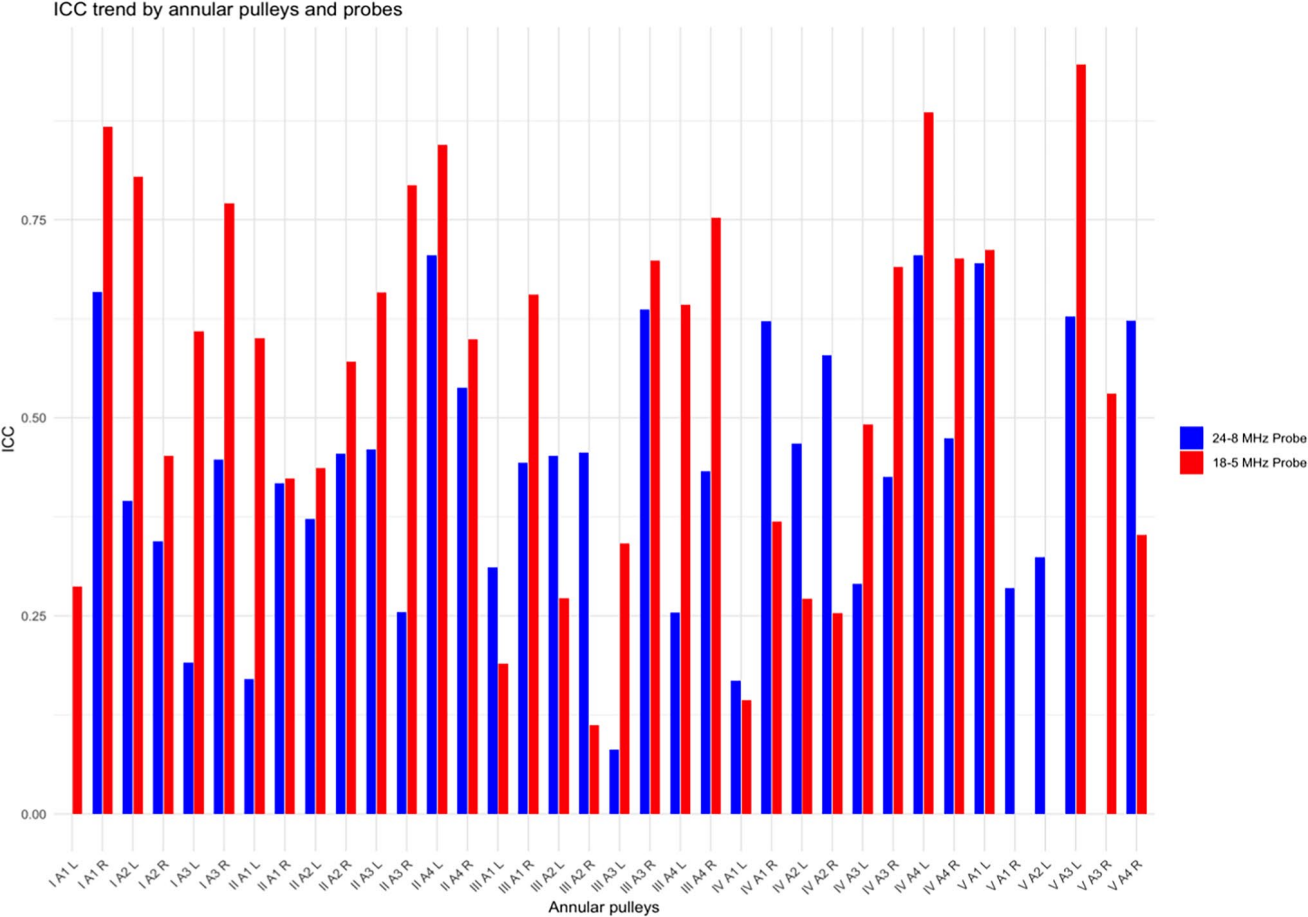
Intraclass correlation coefficient (ICC) between the two operators by annular pulleys and probes type across the toes. The blue bars represent measurements obtained using the 24–8 MHz probe, while the red bars represent measurements obtained using the 18–5 MHz probe. The ICC values vary across the annular pulleys of the toes (I to V) and sides (right [R] and left [L]), indicating differences in measurement reliability between probe types

**Table 4:**
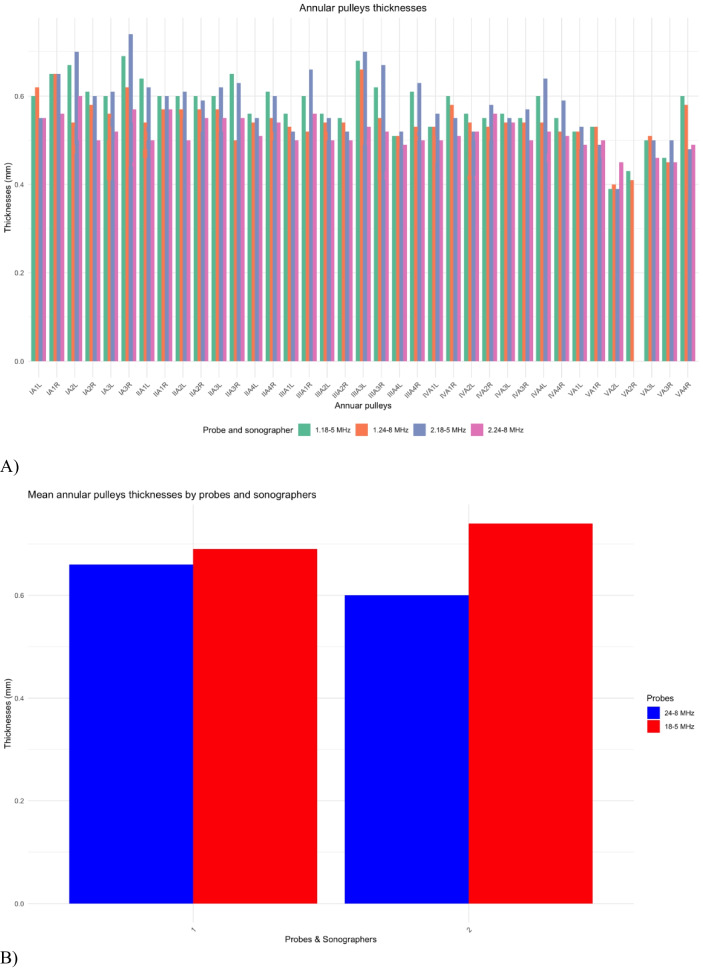
A) Maximal pulley thickness measurements for each toe (I to V) on both sides, right (R) and left (L), obtained with the 18–5 MHz and 24–8 MHz probes, and performed by two different sonographers (1 and 2). The bars represent the mean values for each combination of probe and sonographer, illustrating variability in measurements across toes, sides, and operators. Measurement units are in millimeters (mm). B) Comparison of maximal pulley thickness measurements by probe (18–5 MHz and 24–8 MHz) and sonographer (1 and 2). The bars represent the average measurements in millimeters (mm) for each combination of probe frequency and sonographer

### Pathological cases

#### Case 1

**Fig. 4:**
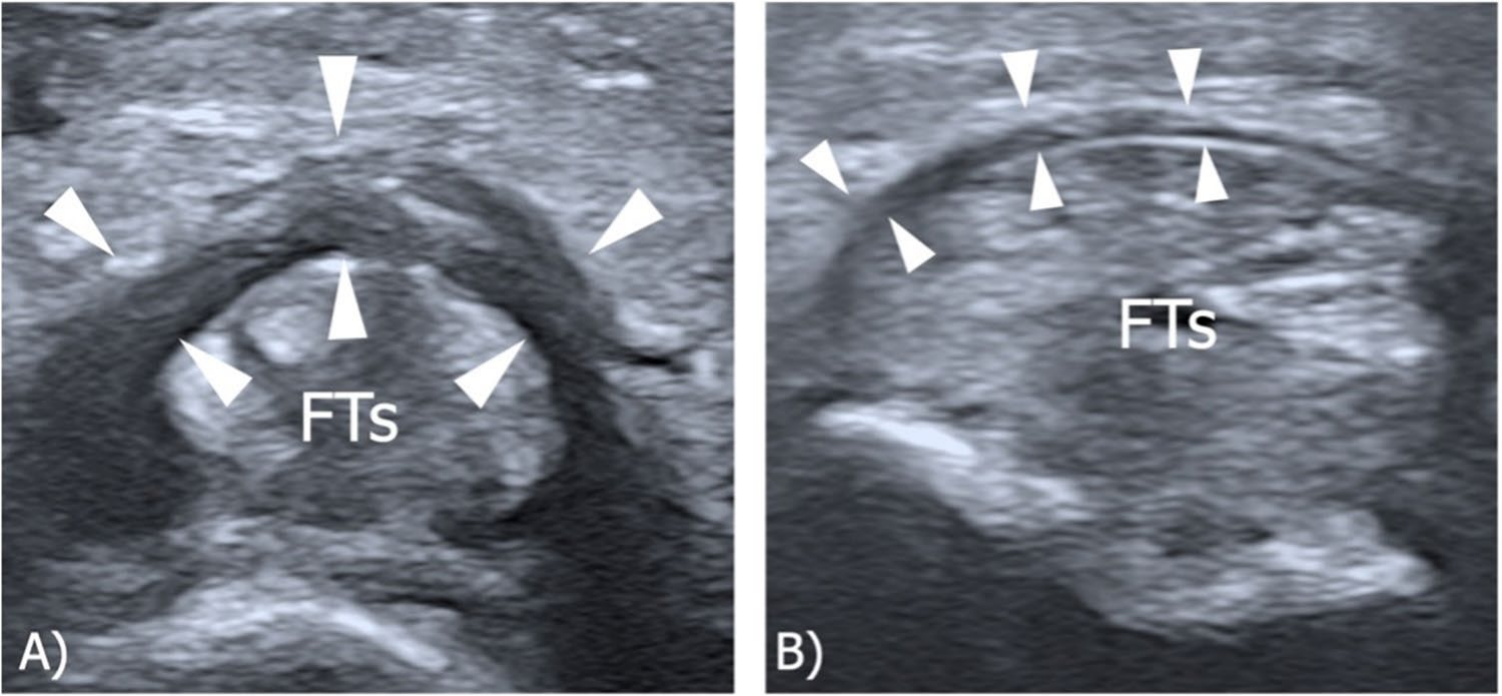
Case 1: Post-traumatic thickening of the annular pulley. Transverse US images of the A3 pulley of the **A** right and **B** left III toe obtained with a 22–8 MHz hockey-stick probe. **A** An inhomogeneous and irregularly thickened A3 pulley (arrowheads) of the III right toe is shown, consistent with a partial annular pulley tear. **B** Note the normal thickness of the A3 pulley (arrowheads) of the left III toe compared to the contralateral one. FTs, flexor tendons

A 60-year-old man was referred by the orthopedics department for a US examination of the foot following a direct trauma to the forefoot. The patient presented with a hammer deformity of the right III toe and impaired flexion of the digit. US imaging did not reveal any continuity lesions of the flexor tendons, but an inhomogeneous A3 pulley of the III toe was noted. In particular, the A3 pulley of the right III toe appeared diffusely irregular and markedly thickened (1.7 mm) compared to the contralateral one (0.56 mm), consistent with an A3 partial tear (Fig. 4). The patient underwent conservatory management with progressive improvement of symptoms.

#### Case 2

**Fig. 5:**
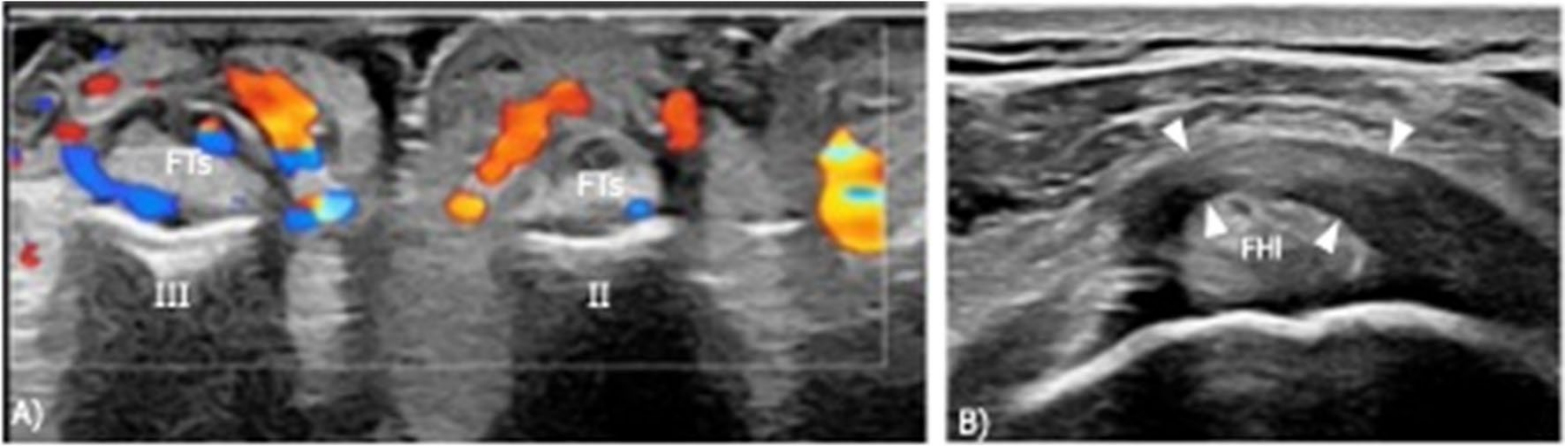
Case 2: Annular pulley thickening in inflammatory arthritis. Transverse US images of the ventral side of right forefoot obtained with a 18–4 MHz probe. **A** Signs of hypervascularization on Doppler imaging around the flexor tendons of the II and III toes. **B** Note the markedly thickened A1 pulley of the great toe (arrowheads). FTs, flexor tendons; FHl, flexor hallucis longus

A 66-year-old man with a clinically suspected spondyloarthropathy underwent US examination to evaluate his swollen and painful right ankle and foot. The ankle US revealed fluid effusion in the subtalar joint and talocalcaneonavicular joint, along with synovial hypertrophy and signs of hypervascularization on Doppler imaging. The sonographic assessment of the foot showed fluid effusion in the dorsal recesses of the II, III, IV, and V metatarsophalangeal joints, as well as diffuse edema of the subcutaneous tissue and signs of hypervascularization on Doppler imaging. Additionally, a markedly thickened A1 pulley (3.1 mm) was noted at the level of the great toe (Fig. 5), which was already affected by onychodystrophy.

#### Case 3

**Fig. 6:**
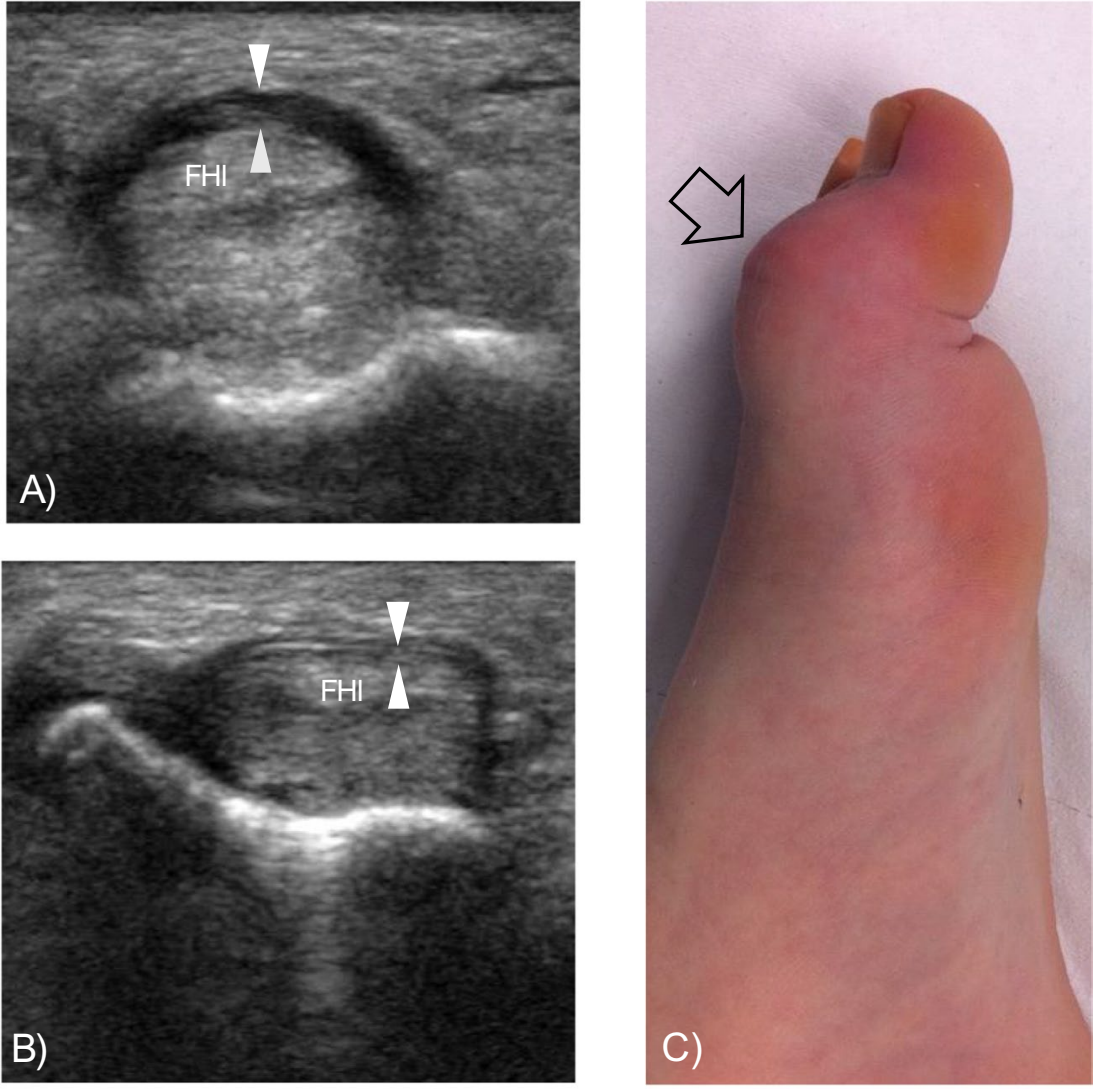
Case 3: Annular pulley thickening in trigger hallux deformity. Transverse US images of the ventral side of the hallux right forefoot obtained with a 17–5 MHz probe. Note the thickening of the A1 pulley (arrowheads) of the **A** right hallux compared to the **B** left hallux. **C** Deformity of the interphalangeal joint (arrow) consistent with a trigger hallux deformity. FHl, flexor hallucis longus tendon

An 18-year-old female ballet dancer was referred for US examination due to the progressive development of a left trigger hallux. As a ballet dancer, the patient spent several hours per week standing on her toes during lessons. Ultrasound examination revealed a significantly thickened A1 pulley of the left hallux (0.8 mm) compared to the corresponding pulley of the unaffected right hallux (0.4 mm) and an impaired gliding of the flexor hallucis longus tendon underneath the A1 pulley during dynamic evaluation, confirming a trigger toe condition (Fig. 6).

## Discussion

This study provides the first description of the sonographic anatomy of the annular pulleys in the flexor tendons of the toes. Consistent with prior cadaveric research, our anatomical and US investigation confirmed the presence of 4 annular pulleys and 2 cruciform pulleys in the lesser toes, as well as 3 annular pulleys and 2 cruciform pulleys in the great toe [1]. These findings slightly differ from the results reported in the MRI study on cadavers by Tafur et al., who described 5 annular pulleys instead of 4 in the lesser toes [2]. This discrepancy could be attributed to variations in anatomical dissections or interpretations of the pulley structures. Furthermore, the difference in spatial resolution between the 11.7 T MR used by Tafur and the high-resolution US employed in our study may have contributed to the observed differences. Notably, Tafur et al. highlighted that the A3 and A5 pulleys were not identified in the cadaveric toe on dissection, whereas they were clearly observed using MRI, probably thanks to the ultrahigh magnetic field. The intensity of the magnetic field likely enabled also the visualization of the cruciform pulleys, which were not clearly distinguishable in our US study. The annular pulleys of the I, II, III, and IV toes could be consistently visualized on US. However, the visualization of the annular pulleys of the V toe appeared less reliable, likely due to the smaller size of the toe, which hindered the maneuverability of the US probe, and the closer proximity between the different pulleys compared to the other toes.

The sonographic appearance of the annular pulleys of the flexor tendons in the toes resembled that of the finger pulleys, presenting as distinct, echogenic bands prone to anisotropy and spanning across the flexor tendon sheath [3].

The measurements obtained by the two operators for each probe demonstrated limited variability, reflecting a moderate inter-operator agreement. The small differences observed might be attributable to slight variations in operator technique or subjective factors inherent to manual measurements. More pronounced variability was noted between the two probes. Certain annular pulleys exhibited larger measurement differences between probes, likely due to variations in frequency range, resolution, and beam focus between the 18–5 MHz and 24–8 MHz probes. Additionally, epidermal thickness of the toes may have further limited US beam penetration, explaining at least in part why the 18–5 MHz probe outperformed the 24–8 MHz probe in this context.

Three indicative pathological cases affecting the toe annular pulleys and resembling the pathology commonly encountered in the annular pulleys of the fingers were reported. Furthermore, in two healthy participants, ganglion cysts were observed near the annular pulleys of the fingers, at the same way of the peri-pulley ganglion cysts commonly encountered in the hand [4, 5]. In the case of partial tear of the toe’s annular pulley, the sonographic appearance was analogous to that observed in the fingers, with irregular thickening of the affected pulley, most evident when compared to the healthy contralateral one [4, 6]. In the reported case of the patient presenting with arthritis of the foot, the evaluation of the annular pulleys demonstrated thickening of an annular pulley of the hallux, similar to observations in the hands of patients with spondyloarthropathy [7]. Then, a case of triggering of the hallux in a female classic ballet dancer was reported. The sonographic appearance of this case of trigger hallux was almost identical to that of a trigger finger, with an asymmetrical thickening of the affected annular pulley compared to the respective contralateral one [8]. This case of trigger hallux aligns with the findings reported by Martin et al., who described toe triggering associated with thickening of the annular pulleys [1]. This mechanism differs from the more common cause of triggering, which is typically localized in the tarsal tunnel beneath the medial malleolus [9–11]. In all reported pathological cases, comparing the thickness of the affected annular pulley with the respective contralateral pulley and the other healthy annular pulleys was crucial for diagnosis.

Although less commonly encountered, pathology of the annular pulleys of the toes does exist and has been described in the literature. One reason it is likely underdiagnosed is the lack of appropriate imaging modalities. While Tafur et al. demonstrated the visualization of toe pulleys using MRI, the machine employed (11.7 T) in their study is not routinely used in clinical practice. Furthermore, no in vivo studies have been previously conducted to validate an imaging modality for evaluating toe pulleys. High-resolution US, as demonstrated in our study, offers an easily accessible and potentially unparalleled modality for evaluating the annular pulleys of the toes. Our study is the first imaging research to demonstrate the annular pulleys in vivo, and despite its limited application in patients, the results appear promising. The clinical implications of our findings could be of note. High-resolution ultrasound US has been proven to be a valuable diagnostic tool for identifying and assessing pathologies of the toe annular pulley, potentially leading to more accurate diagnoses and better treatment planning. The pathological cases presented in our study illustrate the diverse range of conditions that can affect the toe annular pulleys. These cases include partial tears, arthritis-related changes, and triggering phenomena, all of which can be effectively assessed using US. Our findings could also open the door to further research on the prevalence and impact of toe pulley pathologies.

There are two main limitations to our investigation. First, the study involved a relatively limited number of cadaveric specimens (8 feet from 4 cadavers) and a small sample of healthy participants (15 individuals). A larger cohort would provide more robust data and allow for better generalization of the findings. Second, we reported only three pathological cases, which probably do not fully represent the range of possible pathologies affecting the annular pulleys of the toes. In conclusion, our study provides a comprehensive description of the sonoanatomy of the annular pulleys of the flexor tendons in the toes and demonstrates the potential of high-resolution US as a valuable tool for diagnosing and assessing pathologies of these structures. The pathological cases reported underscore the clinical relevance of our findings and suggest that toe pulley pathologies, although less commonly recognized, warrant further attention.

## Data Availability

The datasets generated and analyzed during this study are not publicly available due to privacy and ethical restrictions. However, they can be obtained from the corresponding author upon reasonable request and with approval from the institutional review board.
